# The dynamics of vertebrate homeobox gene evolution: gain and loss of genes in mouse and human lineages

**DOI:** 10.1186/1471-2148-11-169

**Published:** 2011-06-16

**Authors:** Ying-fu Zhong, Peter WH Holland

**Affiliations:** 1 Department of Zoology, University of Oxford, South Parks Road, Oxford, OX1 3PS, UK

## Abstract

**Background:**

Homeobox genes are a large and diverse group of genes, many of which play important roles in transcriptional regulation during embryonic development. Comparison of homeobox genes between species may provide insights into the evolution of developmental mechanisms.

**Results:**

Here we report an extensive survey of human and mouse homeobox genes based on their most recent genome assemblies, providing the first comprehensive analysis of mouse homeobox genes and updating an earlier survey of human homeobox genes. In total we recognize 333 human homeobox loci comprising 255 probable genes and 78 probable pseudogenes, and 324 mouse homeobox loci comprising 279 probable genes and 45 probable pseudogenes (accessible at http://homeodb.zoo.ox.ac.uk). Comparison to partial genome sequences from other species allows us to resolve which differences are due to gain of genes and which are due to gene losses.

**Conclusions:**

We find there has been much more homeobox gene loss in the rodent evolutionary lineage than in the primate lineage. While humans have lost only the *Msx3 *gene, mice have lost *Ventx, Argfx, Dprx, Shox, Rax2, LOC647589, Tprx1 *and *Nanognb*. This analysis provides insight into the patterns of homeobox gene evolution in the mammals, and a step towards relating genomic evolution to phenotypic evolution.

## Background

Homeobox genes comprise a large and diverse group of genes, most of which are thought to act as transcription factors. They are characterised by possession of one or more homeobox sequences of 180 base pairs (or longer) encoding homeodomain peptides that fold into helix-loop-helix-turn-helix domains [1]. Homeobox genes are found across eukaryotes but are most diverse in animal genomes, following an evolutionary expansion of this group of genes in the early evolution of Metazoa [2]. The best known homeobox genes are Hox genes, usually arranged into gene clusters and known to play pivotal roles in specification of cell identity along the developing anteroposterior body axis in the embryos of bilaterian animals. Many other homeobox genes also have key roles in animal development, with homeobox genes implicated in development of the brain, central nervous system, skeleton, muscle, neural crest-derived tissues, appendages, heart, liver and other structures.

After many years of confusion and debate, a robust evolutionary classification of metazoan homeobox genes has been established. Although there are slight differences between authors, most schemes recognise 11 'classes' of homeobox genes, within which there are over 100 gene families [3-5]. Gene families are defined as all genes descended from a single progenitor gene in the common ancestor of bilaterian animals, although some additional gene families have been erected for genes of unknown orthology. Having a robust classification of gene facilitates comparison between species. There has been much interest in elucidating the evolutionary history of homeobox genes, partly to ascertain whether evolutionary changes in animal body organisation are reflected in changes to the homeobox gene repertoire of animals. It is known, for example, that the emergence of vertebrates from their invertebrate chordate ancestors was accompanied by an expansion in the number of homeobox genes, from around 100 to over 200, through genome duplication plus retention of duplicated genes [6]. Far less has been documented about the dynamics of homeobox gene evolution within the vertebrates, although several studies focussed on individual genes or gene families have highlighted occasional gene duplications and gene losses.

To investigate the evolutionary dynamics of the complete homeobox gene superclass in mammalian genomes, we chose to focus on two species whose genomes have been sequenced to high coverage, accurately assembled and reasonably well annotated: human and mouse. The comparison is additionally useful because the biological functions of many human homeobox genes were inferred through analysis of orthologues in mouse. Differences between any two species, however, cannot be interpreted in an evolutionary way without reference to other genomes. Our aim, therefore, was to characterise the similarities and differences between human and mouse homeobox gene repertoires, and then resolve these into gene losses or gene gains on either the primate or rodent lineages by comparison to the genomes of other vertebrate species. A comprehensive survey of homeobox loci in the human genome was undertaken by Holland et al. (2007) [3], using genome sequence Build 35.1. This survey listed 300 human homeobox loci comprising 235 probable functional genes and 65 probable pseudogenes. In the intervening years the human genome sequence has been refined, with the most recent assembly being Build 37.2 (GRCh37.p2) released by the Genome Reference Consortium in 2010 [7]. We have therefore used this opportunity to update the survey of human homeobox genes. Mouse, being a widely used model species for biological and biomedical research, was an early target for genome sequencing [8]. The most recent version of the mouse genome assembly is Build 37 (MGSCv37, C57BL/6J), produced by the Mouse Genome Sequencing Consortium (MGSC) [9]. The complete homeobox gene repertoire of the mouse genome has not been analysed previously.

Here we report a systematic identification of human and mouse homeobox genes. Comparison between these species and other vertebrates reveals all homeobox gene gains, gene duplications and gene loss events that occurred during the evolution of the two species since they diverged from a common ancestor. We find that the extent of homeobox gene duplication and the extent of gain of novel, divergent genes has been similar in the two evolutionary lineages; however, there has been much more homeobox gene loss in the ancestry of rodents than in the ancestry of humans.

## Results

### Human homeobox gene repertoire

We identified 333 homeobox loci in the human genome, including 255 probable functional genes and 78 probable pseudogenes (Table 1; Additional File 1). Compared to an earlier survey based on a previous assembly of the human genome [3], 33 new loci were detected in the present study.

**Table 1:** Classification of homeobox genes in human and mouse genomes

Class	Number of Gene Families		Number of Genes		Number of Pseudogenes	
	
	Human	Mouse	Human	Mouse	Human	Mouse
**ANTP**	37	36	100	100	19	2

**PRD**	31	28	66	87	32	30

**LIM**	6	6	12	12	0	0

**POU**	7	7	16	16	8	0

**HNF**	2	2	3	3	0	0

**SINE**	3	3	6	6	0	0

**TALE**	6	6	20	22	10	1

**CUT**	3	3	7	7	3	0

**PROS**	1	1	2	2	0	0

**ZF**	5	5	14	14	1	0

**CERS**	1	1	5	5	0	0

**(other)**	2	4	4	6	5	11

**Totals**	**104**	**102**	**255**	**279**	**78**	**45**

**Figure 1:**
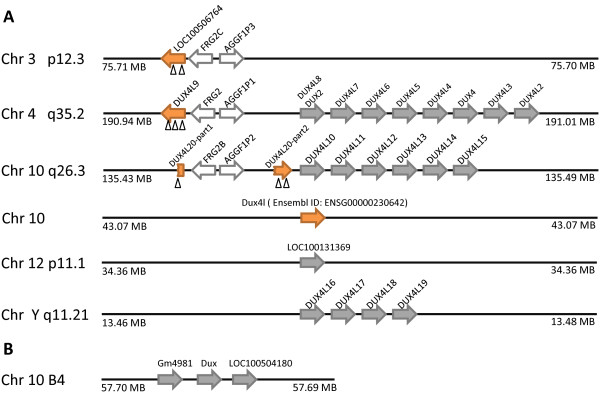
**Chromosomal distribution of human *DUX4-*like genes and mouse 'chromosome 10' Dux Genes**. (A) Twenty-three human *DUX4*-like sequences include four loci with predicted introns interrupting the coding sequence (orange arrows) and nineteen intronless sequences (grey arrows), most clustering on chromosomes 4, 10 and Y. One putative intron-containing locus at chromosome 10q26.3 is disrupted by a translocation or is the remnants of two loci. (B) The Dux gene on mouse chromosome 10 has been duplicated in tandem to generate three loci. Orientation of arrows indicates direction of transcription; small arrowheads indicate putative introns.

**Figure 2:**
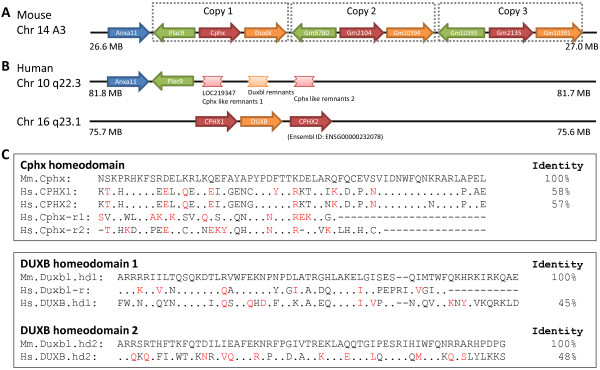
***DUXB *and *CPHX *loci in mouse and human**. (A) Mouse *DUXB-like *(*Duxbl*, orange arrows) and *Cphx *(red arrows), together with a non-homeobox gene *Plac9 *(green arrows), have been duplicated giving three copies each at chromosome 14A3. (B) Human *Cphx*-related homeobox loci flank a *DUXBL *pseudogene and the *DUXB *gene on chromosomes 10 and 16 respectively, although the loci on chromosome 10 are disrupted (ragged boxes). *Anxa11 *(blue arrows) is not a homeobox genes. (C) Amino acid alignments; dots represent identical amino acids, dashes deletons/insertions and red characters conservative substitutions. Hs, *Homo sapiens*; Mm, *Mus musculus*.

Of these 33 loci, 24 are members of the Dux (double homeobox) family. The evolution of this gene family is complex because the homeobox sequence, or sequences, of an ancient Dux sequence have become incorporated into repetitive DNA elements ('DUX4' sequences) found in both heterochromatin and euchromatin. It is also possible that these sequences vary in copy number between individuals. The 24 newly identified human Dux loci include 23 '*DUX4' *sequences (Figure 1) and a degraded sequence related to *DUXBL *(Figure 2). The 23 *DUX4*-like sequences include four loci with putative introns interrupting the coding sequence (on chromosomes 3, 4 and 10), although one of these loci (on chromosome 10) is disrupted by a translocation or alternatively is the remnants of two degraded pseudogenes (Figure 1). Most of the *DUX4*-like sequences are intronless and arranged in arrays; these include the D4Z4 locus at 4q35 linked to facioscapulohumeral muscular dystrophy [10,11]. In this array, one sequence is identical to the original *DUX4 *sequence initially reported as an isolated clone but not previously found in a genomic assembly.

Following the first report of an intron-containing Dux gene on chromosome 19 (*DUXA*) by Booth and Holland (2007) [12], a distinct intron-containing locus *DUXB *on chromosome 16 was reported by Holland et al. (2007) [3]. Clapp et al. (2007) [13] found that two further intron-containing Dux genes, *DUXC *and *DUXBL *(*DUXB-like*), were present in some mammals but lost from human, although subsequent work from the same research group showed that a degraded *DUXBL*-related sequence does exist in the human genome at chromosome 10q22 in a syntenic region to the mouse *Duxbl *locus located at 14 A3 [14]. Leidenroth and Hewitt (2010) [14] showed that the *DUXBL *sequence at 10q22 is truncated (it lacks the second homeobox sequence) and is most likely a pseudogene. The same authors identified local paralogy between the human *DUXB *and *DUXBL *locations, as both are adjacent to a *Cphx*-like sequence, indicative of a possible segmental duplication followed by transposition. Our analyses confirm these findings, but detect additional *Cphx-loci*. We find that these flank both *DUXB *and *DUXBL *providing additional support for segmental duplication in the human genome (Figure 2).

The Cphx (cytoplasmic polyadenylated homeobox) gene family was first described from mouse [15]. No orthologue was described in the survey of Holland et al. (2007) [3], although subsequently the Mouse Genome Informatics (MGI) Mammalian Orthology and Comparative Mapping applications identified human LOC219347 on chromosome 10 as the putative human *CPHX *gene, and the Ensembl project identified a locus on chromosome 16 as another putative human *CPHX *gene (Ensemble ID: ENSG00000232078). Our analyses of genome build 37.2 reveal the situation is more complex. We find four human *Cphx*-related homeobox loci on chromosomes 10 and 16, flanking the *DUXBL *pseudogene and the *DUXB *gene (Figure 2). Although these sequences have only weak sequence similarity to the *Cphx *homeobox loci of mouse (maximally 35/60 residues) they are in syntenic locations (or one syntenic location plus one duplicated region) highly suggestive of cryptic orthology. The two *Cphx*-like loci at 10q22.3 are both truncated, with frameshifts in the sequence encoding helix 3 of the homeodomain; we classify these as pseudogenes. The two *Cphx*-like loci at 16q23.1 (*CPHX1 *and *CPHX2*) have complete homeobox sequences and possess introns; these loci may be functional.

**Figure 3:**
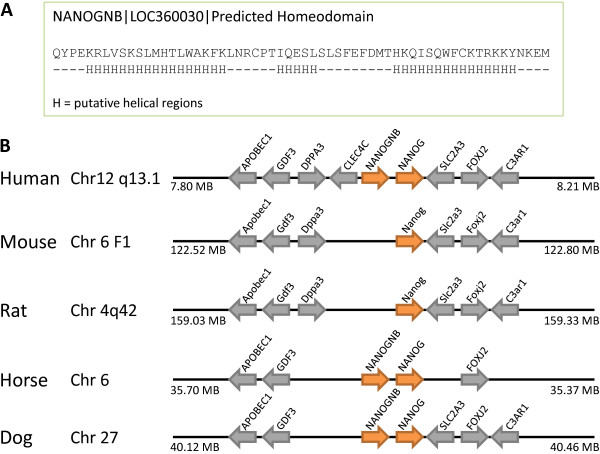
**Presence or absence of the *NANOGNB *gene in mammalian genomes**. (A) The deduced homeodomain sequence of human *NANOGNB *showing predicted alpha helical regions, compatible with folding into a homeodomain tertiary structure. (B) *NANOGNB *is located just 15 kb from the human *NANOG *gene at chromosome 12p13.31 (orange arrows). Orthologous genes at the syntenic position are present in horse and dog, but not in mouse and rat. Grey arrows indicate non-homeobox genes.

Four additional loci included in the current survey define a new gene family: Nanognb. The single putative functional member of this gene family in human is the *NANOGNB *(*Nanog *neighbour) gene, a locus located only about 15 kb from the human *NANOG *gene at chromosome 12p13.31 (Figure 3). The locus, labelled as EntrezGene LOC360030, was originally named 'homeobox C14' but this name has been changed to *NANOGNB *by the Human Gene Nomenclature Committee to avoid confusion with Hoxc cluster genes which are not closely related. The sequence was excluded from the homeobox gene survey of Holland et al. (2007) [3] due to its overall weak similarity to other homeobox genes; however, further analysis reveals that the deduced homeodomain sequence, though divergent, would be capable of folding into three alpha helices, compatible with a homeodomain tertiary structure. Three new loci were detected that are pseudogenes derived from *NANOGNB*, located on chromosomes 2, 12 and X, whereas the original locus at 12p13 contains a complete homeobox sequence and possible introns. Together these four loci define a gene family that cannot be accommodated easily into one of the 11 established gene classes of metazoan homeobox.

**Figure 4:**
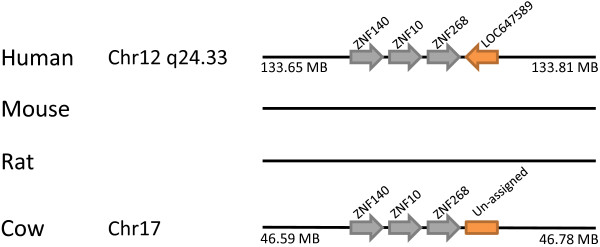
**Absence of *LOC647589 *in the mouse genome**. *LOC647589 *was detected in this study at human chromosome 12q24.33. No annotation is found in genomes outside of human currently, although a homologous sequences is present in cow (chr17:46,768,917-46,783,902) and other mammals with available genome sequences, but not in mouse or rat. The orange arrow and box indicate homeobox loci; grey arrows indicate non-homeobox genes.

The final additional gene found in the present survey is LOC647589 at chromosome 12q24.33 (Figure 4). It cannot be accommodated easily into established gene classes and families.

### Mouse homeobox gene repertoire

In the mouse genome, we identified 324 homeobox loci, which we divide into 279 probable functional genes, 45 probable pseudogenes.

As in the human genome, most homeobox genes are scattered on different chromosomes, with a minority clustered together. The Hoxa, Hoxb, Hoxc and Hoxd gene clusters are well known and identical in gene complement between human and mouse. In contrast, the Dux clusters and Rhox cluster are very different in composition between the two species, and the Obox gene cluster is present in mouse but not human.

The Dux (double homeobox) cluster is complex, and its evolution has been studied in detail by Clapp et al. (2007) [13]. In mouse, we identify six Dux loci: Gm4981, *Dux *and LOC100504180 at chromosome 10 B4 (Figure 1), and *Duxbl *and its two tandem copies (Gm10394 and Gm10391) at chromosome 14 A3 [16]. It is interesting to note that *Duxbl*, together with another homeobox gene *Cphx*, has been tandemly duplicated twice giving three copies each of *Duxbl *and *Cphx *(Figure 2). This triplication event is not detected in other mammalian genomes, even in rat, suggesting it occurred recently in evolution.

**Figure 5:**
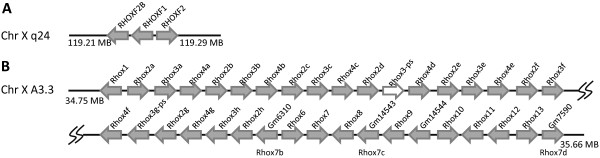
**Rhox (reproductive homeobox) gene clusters**. (A) Three Rhox loci are present in the human genome at chromosome Xq24. (B) Thirty-six Rhox loci are clustered in the mouse genome at chromosome X A3.3. *Rhox3-ps *(white arrow) has a stop codon in the homeobox.

Rhox (reproductive homeobox) genes, are expressed during embryogenesis and gametogenesis [17]. In the mouse genome, we identified 36 Rhox loci. All Rhox loci we indentified are clustered together at chromosome X A3.3 (Figure 5), but it should be noted that the previously reported *Rhox5 *is not included in the reference assembly genome. As noted by others [18], the Rhox gene cluster is larger than most other homeobox gene clusters. In contrast, the human genome sequence includes only three Rhox genes found located at chromosome Xq24 (Figure 5).

The full repertoire of mouse homeobox genes is summarized in Table 1 and described in Additional Files 2, 3 and 4.

### Origin of a homeobox gene on the primate lineage

Genes that are present in one species but not another could reflect either gene loss in one lineage or the origin of a new gene (usually by duplication and divergence) in the other lineage. These two mutually exclusive possibilities can be distinguished clearly by examining other genomes, taking into account the phylogenetic history between the various species. Using this approach, we deduce that just one homeobox gene in the human genome is a novel gene that arose during primate evolution since the divergence of the primate and rodent evolutionary lineages.

**Figure 6:**
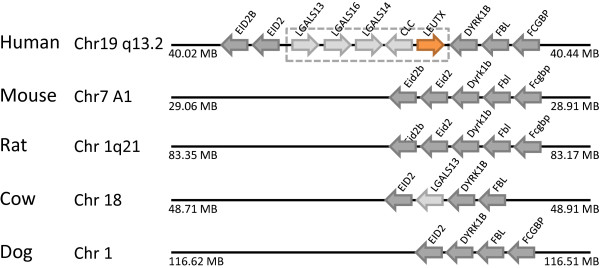
**Origin of the *LEUTX *gene in the human genome**. The *LEUTX *homeobox gene (orange arrow) plus four unusual non-homeobox genes (light grey arrows) form a linked set of genes in the human genome (dashed box). These genes are not present at the syntenic location in mouse, rat, cow or dog genomes.

*LEUTX *(leucine twenty homeobox) is a gene of unknown function that is present in the human genome at position 19q13.2 (Figure 6; [3]. Closely related sequences can be detected in the draft genome sequences of other primates (chimpanzee, orangutan, rhesus macaque), and these are located at a genomic location syntenic to human *LEUTX *(Table 2; Additional File 5). No homologues are detected outside primates. Examination of the syntenic region in mouse, rat, cow and dog reveals absence of the gene at the expected location and presence of a set of unusual genes in human (Figure 6). We suggest therefore that *LEUTX *arose on the primate lineage. It has been hypothesized previously that *LEUTX *arose by tandem duplication and extreme divergence from the Otx family gene *CRX *which is located on the same chromosome (8 MB distant), although timing was unknown [3].

**Table 2:** Taxonomic distribution of homeobox genes differing between human and mouse genomes

Species	Human	Chimpanzee	Macaque	Mouse	Rat	Cow	Pig	Horse	Dog	Chicken	Frog
***Leutx***	P	P	P	-	-	-	-	-	-	-	-

***Obox***	-	-	-	P	P	-	-	-	-	-	-

***Crxos1***	-	-	-	P	P	-	-	-	-	-	-

***Gm5585***	-	-	-	P	P	-	-	-	-	-	-

***Gm7235***	-	-	-	P	P	-	-	-	-	-	-

***Msx3***	-	-	-	P	P	-	P	P	-	-	-

***Ventx***	P	P	P	-	-	-	-	-	P	P	--

***Argfx***	P	P	P	-	-	P	-	P	-	-	-

***Dprx***	P	P	P	-	-	P	-	P	P	-	-

***Shox***	P	P	P	-	-	P	P	-	P	P	P

***Rax2***	P	P	P	-	-	P	-	-	P	P	P

***LOC647589***	P	P	P	-	-	P	P	P	P	-	-

***Tprx1***	P	P	P	-	-	-	-	-	P	-	-

***Nanognb***	P	P	P	-	-	-	-	P	P	-	-

P: Present, as inferred by greater than 90% amino acid identity over homeodomain or a lower identity but with same synteny. -: Not present, as inferred by no sequence with 90% amino acid identity or no similar gene in syntenic location.

### Origin of homeobox genes on the rodent lineage

Using the same comparative approach, we deduce that the Obox homeobox gene cluster, plus three other homeobox genes, arose during on the evolutionary lineage leading to mouse since the divergence of the primate and rodent evolutionary lineages.

**Figure 7:**
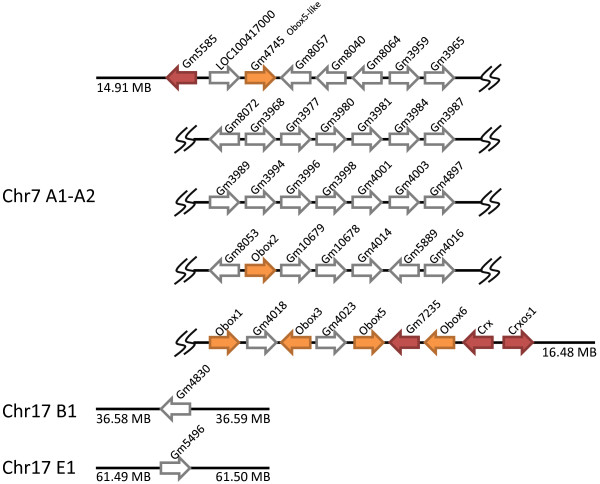
**The mouse Obox loci and associated genes**. There are 36 mouse Obox homeobox loci, comprising 6 intron-containing genes (orange arrows) and 28 intronless loci (unfilled arrows) on chromosome 7, plus intronless probable pseudogenes on chromosome 17. Four other homeobox genes, not clearly part of the Obox family, are linked to the large Obox cluster: *Crxos1, Gm5585 *and *Gm7235 *and *Crx *(red arrows).

The *Obox *(oocyte specific homeobox) genes have been reported previously [19] and implicated in reproductive biology of the mouse, but the full complexity of this gene family is not widely appreciated. We identified 36 Obox family loci in total, including one large cluster at chromosome 7 A1-2 regions (6 intron-containing genes and 28 intronless loci) and two intronless probable pseudogenes at chromosome 17 B1 and E1 (Figure 7). The previously reported *Obox4 *gene is not included here since it is absent from the assembled genome sequence. Transcripts from the Obox gene family are preferentially detected in the gonads [19] although high-throughput screens have detected expression in additional tissues [20,21]. Since clear homologues of Obox genes are not present in primates, nor other mammals, we suggest that the *Obox *gene family emerged specifically in rodents after the divergence of primates and rodents with further multiple duplications resulting in the tandem organization of *Obox *in murine genomes [22].

Three further homeobox genes, which we have only found in rodents thus far, are located physically close to - or within - the Obox gene cluster. *Crxos1 *is a mouse homeobox gene expressed in murine embryonic stem (ES) cells and is essential for ES cell self-renewal [23]. It is located at chromosome 7 A1, adjacent to the *Crx *gene, on the opposite side to the Obox genes (Figure 7). *Crxos1 *contains 5 exons and 4 introns, and encodes two homeodomain sequences which cannot readily be classified into any of the eleven main homeodomain classes. Eight pseudogenes are also found that contain one of the two *Crxos1 *homeodomains. In addition there are 10 unannotated homeobox loci with lower similarity to *Crxos1*, ranging from 68% to 84% similarity over region of homeodomain (Additional File 4). Gm5585 and Gm7235 are two other homeobox loci present only in rodents according to our database searches. Gm5585 has predicted introns, and Gm7235 has an unclear gene structure. All three loci have homeobox sequences that are quite distinct from the Obox genes, but they are close physical proximity (Figure 7). It is possible, therefore, that they originated in the same series of tandem duplications that generated the Obox gene cluster, followed by sequence divergence. We also detected three pseudogenes of Gm5585 at chromosome 2B, 9F1 and XE1.

### Loss of homeobox genes

As outlined above, genes that are present in one species but not another could reflect either gene loss or gene gain. We can infer that gene loss has taken place if a range of mammalian species possess the gene, which is missing in just one mammalian lineage (human or mouse). This inference is particularly solid if the gene is present in an evolutionary lineage that is a phylogenetic outgroup to primates plus rodents, or in an ingroup lineage that is related to the species lacking the gene in question. Using this logic, we infer that one homeobox gene was lost from the human genome and eight were lost from mice, since the divergence of these two evolutionary lineages (Table 2).

**Figure 8:**
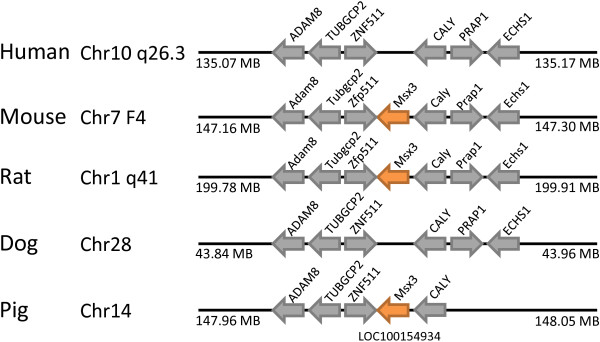
**The *MSX3 *gene has been lost in human and dog genomes**. The *Msx3 *gene is located in syntenic regions of mouse, rat and pig genomes (orange arrows), but absent at the equivalent location in human and pig. Non-homeobox genes used as indicators of chromosomal synteny are shown as grey arrows.

The sole homeobox gene deduced to have been lost in human, compared to mice, is the *MSX3 *(muscle segment homeobox 3) gene. The *Msx3 *gene is a member of Msx family within the ANTP class and was first reported in mouse [24]. This gene is present in the mouse and rat genomes, but is absent from the syntenic position in human (Figure 8). Through phylogenomic analysis, we found a related sequence (LOC100154934) in the pig genome in the syntenic genomic region. Furthermore, it has been shown previously that the *Msx3 *genomic region forms part of a fourfold paralogy group with *Msx1 *and *Msx2*, arising in the genome duplications in early vertebrate evolution [25]. It is clear, therefore, that *Msx3 *was lost secondarily in primate evolution.

**Figure 9:**
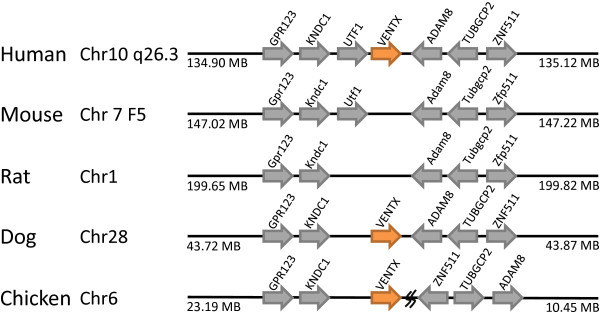
**The *Ventx *gene has been lost in rodent genomes**. The *VENTX *gene is located in syntenic regions of human, rat, dog and chicken genomes (orange arrows), but absent at the equivalent location in mouse and rat. Non-homeobox genes used as indicators of chromosomal synteny are shown as grey arrows.

In contrast to this single gene loss, the mouse genome has secondarily lost *Ventx, Argfx, Dprx, Shox, Rax2, LOC647589, Tprx1 *and *Nanognb *since divergence from the common ancestor of rodents and primates. The *Ventx *(VENT homeobox) gene is found at human chromosome 10q26.3. There are two tandemly arranged putative orthologues in amphioxus, with identical homeodomains to each other [26], plus clear orthologues on chicken chromosome 6 and dog chromosome 28, at syntenic positions to the human gene (Figure 9). Presence in chicken, and possibly amphioxus, indicates that the gene is older than mammals. No *Ventx *gene can be found in the mouse or rat genomes, although the syntenic region is found at mouse chromosome 5B3 and on rat chromosome 1, clearly indicating gene loss (Figure 9).

**Figure 10:**
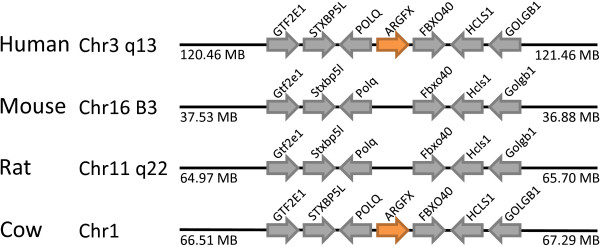
**The *ARGFX *locus has been lost in rodent genomes**. The *ARGFX *gene is located in syntenic regions of human and cow (orange arrows), but absent at the equivalent location in mouse and rat. Non-homeobox genes used as indicators of chromosomal synteny are shown as grey arrows.

The *Argfx *(arginine-fifty homeobox) gene was first detected in the human genome [12] and related loci, in a syntenic position, have since been reported from many other mammals albeit with critical sequence changes that disrupt the deduced coding region [27]. The gene has clearly been lost from rodent genomes [27] (Figure 10).

**Figure 11:**
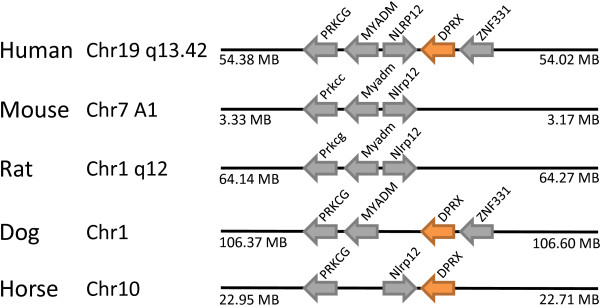
**The *DPRX *gene has been lost in rodent genomes**. The *DPRX *gene is located in syntenic regions of human, dog and horse (orange arrows), but absent at the equivalent location in mouse and rat. Non-homeobox genes used as indicators of chromosomal synteny are shown as grey arrows.

The *Dprx *(divergent-paired related homeobox) gene was also first found in the human genome, with the putative functional locus at 19q13 and seven dispersed pseudogenes [12]. In the present study we found orthologues in the genomes of dog and horse, but not mouse or rat, at the syntenic region (Figure 11). Since dog and horse belong to the Laurasiatheria lineage, while mouse, rat and human are Supraprimates, this is clearly indicative of gene loss in rodents.

**Figure 12:**
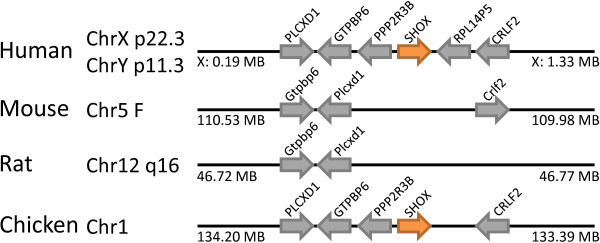
**The *SHOX *gene has been lost in rodent genomes**. The *SHOX *gene is located in syntenic regions of human (X and Y chromosomes) and chicken (orange arrows), but absent at the equivalent location in mouse and rat. Non-homeobox genes used as indicators of chromosomal synteny are shown as grey arrows.

The Shox (short stature homeobox) gene family includes *SHOX *and *SHOX2 *in the human genome, but only *Shox2 *in the mouse genome. Interestingly, in humans the *SHOX *gene is present on both X and Y chromosomes, presumably descendent from a formerly autosomal region, while *SHOX2 *is on chromosome 3. The duplication between *Shox *and *Shox2 *is ancient, since chicken has a *Shox *gene in a syntenic position to the human gene (Figure 12). The absence in mouse, in the equivalent genomic context, is indicative of gene loss.

**Figure 13:**
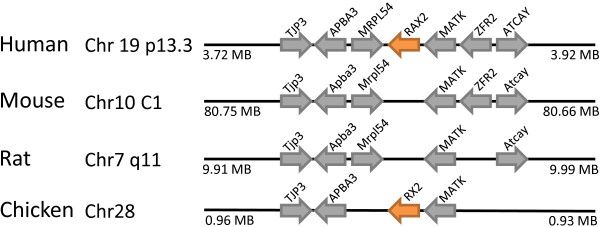
**The *RAX2 *gene has been lost in rodent genomes**. The *RAX2 *gene is located in syntenic regions of human and chicken (orange arrows), but absent at the equivalent location in mouse and rat. Non-homeobox genes used as indicators of chromosomal synteny are shown as grey arrows.

The Rax (retina and anterior neural fold homeobox) gene family includes *RAX *and *RAX2 *in the human genome, but only RAX in the mouse genome. We found orthologues of *RAX2 *in the chicken genome, but not in the syntenic region of mouse or rat, clearly indicating gene loss (Figure 13).

As noted earlier, LOC647589, located at chromosome 12q24.33 in human, was first detected in this survey (Figure 4). No annotation is found in genomes outside of human currently, although we find that homologous sequences can be detected in dog (chr26:3,013,605-3,024,652), cat (scaffold_2339:32,127-43,778), cow (chr17:46,768,917-46,783,902), pig (chr14:22,079,384-22,092,812) and horse (chr8:30,059,056-30,073,471) genome sequences, although not in mouse or rat. This phylogenetic distribution dates the origin of the gene to before the divergence of Supraprimates (including human and mouse) and Laurasiatheria (including dog, cat, cow, pig and horse). Examining its genomic context, we found that the gene is linked to a series of genes encoding zinc finger proteins in the human and cattle genomes; mouse and rat are lacking this genomic region, indicating gene loss (Figure 4).

**Figure 14:**
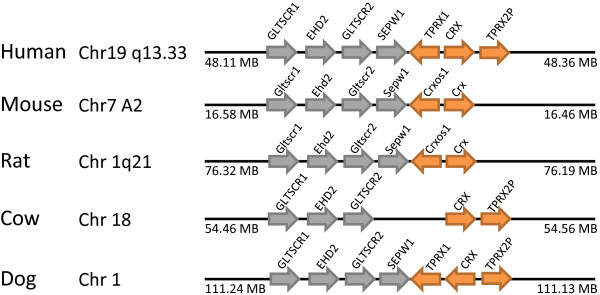
**Tprx loci are not present in rodent genomes**. In the human genome, the Tprx family contains one probable functional gene (*TPRX1*) and a possibly non-functional tandem duplicate (*TPRX2P*) at chromosome 19q13, either side of the Otx-family *CRX *gene. The additional Tprx loci, *TPRX1P1, TPRX1P2 *and *TPRXL, *are elsewhere in the genome and are not shown. Examination of the region syntenic to human 19q13 in other mammals reveals clear orthologues of Tprx family loci in cow and dog, but not mouse and rat. In rodents, another homeobox gene *Crxos1 *is found. Orange arrows indicate homeobox genes; grey arrows are non-homeobox genes.

The Tprx (tetra-peptide repeat homeobox) gene family contains one probable functional gene in human (*TPRX1*), one tandem duplicate that is possibly non-functional (*TPRX2P*), two retrotransposed pseudogenes (*TPRX1P1, TPRX1P2*) and one unusual, probably non-functional, expressed sequence (*TPRXL*). The *TPRX1 *locus is incorrectly annotated in the NCBI and EBI assemblies, as cDNA clone data indicate an additional 5' exon and a different homeobox sequence (Additional File 6). The *TPRX1 *and *TPRX2P *loci flank the *CRX *gene in human. Examining the syntenic region in other mammalian species reveals a complex picture (Figure 14). In dog, both *TPRX1 *and *TPRX2P *are present, as is the latter locus in cow. We infer, therefore, that this condition predates the divergence of the Supraprimates (including human and mouse) and Laurasiatheria (including dog and cow) lineages of placental mammals. This implies loss in rodents. However, it is noteworthy that the same genomic region in rodents contains the *Obox *and *Crxos1 *loci (Figures 7, 14) and is currently unclear if these genes arose separately on the rodent lineage or by duplication and divergence from Tprx loci.

As noted above, *NANOGNB *(LOC360030) is located close to the *NANOG *gene but is highly divergent from it. Orthologous genes at the syntenic position are present in horse and dog indicating that the gene predates the divergence of the Supraprimates and Laurasiatheria (Figure 3). Absence in mouse and rat is therefore a secondary condition.

## Discussion

We identified and classified 324 homeobox loci in the mouse genome. For completeness, these include many loci that are probably pseudogenes that do not have potential to code for functional proteins. Our current estimate of the number of functional homeobox genes in the mouse is 279. Although most mouse homeobox loci are dispersed around the genome, there are several large genomic clusters or arrays. These include the four well-known Hox gene clusters, plus the more recently characterised and much larger Obox and Rhox clusters. Our analyses have refined the structure of the latter two clusters, revealing some hitherto undescribed loci, plus some novel divergent homeobox loci that are in, or close to, the Obox array.

To enable accurate comparison between species, we have refined the human homeobox survey of Holland et al. (2007) [3] using more recent genome sequence and assembly data. The principle changes made are an updated and enlarged survey of human Dux family sequences and genes, inclusion of the *NANOGNB *gene (formerly C14) and its pseudogenes, identification of four Cphx family loci and inclusion of the newly annotated locus LOC647589. These updates, together with the mouse loci, have been incorporated into the latest on-line release of HomeoDB [28], freely accessible at http://homeodb.zoo.ox.ac.uk.

**Figure 15:**
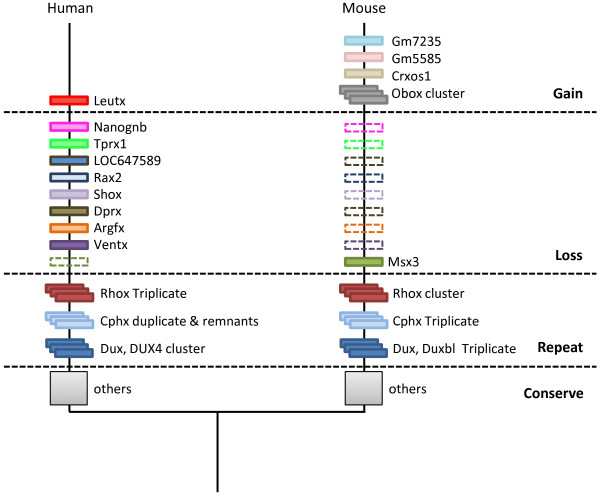
**A summary of homeobox gene dynamics in the mouse and human evolutionary lineages**. The majority of homeobox genes are conserved between mouse and human lineages (grey squares), although some have undergone duplication to different extents (cascaded boxes). Humans have lost the *Msx3 *gene; mice have lost *VENTX, ARGFX, DPRX, SHOX, RAX2, LOC647589, NANOGB *and *TPRX1 *(dashed boxes). Three new homeobox loci (*Gm7235, Gm5585 *and *Crxos1*) and one new cluster (Obox) arose in the rodent lineage; one new gene *Leutx *arose in the lineage leading to primates.

A simple comparison between the surveys of mouse and human homeobox loci highlights the higher number of homeobox loci found in the mouse genome, although this figure is heavily influenced by the extensive Obox and Rhox arrays. More meaningful insight is gained by considering each gene family in turn, and assessing whether differences represent gain or loss in one or other evolutionary lineage. This was undertaken by focussing on each difference between the two species and examining the genomes of other mammals. Ideally, such a comparison would use completely sequenced, assembled and annotated genome sequences from multiple species, but this is yet possible with current datasets. Instead, we exploited the fact that mammalian genomes, and to a lesser extent the genomes of other vertebrates, show extensive synteny, so that genomic regions harbouring a particular homeobox locus in either mouse or human could be searched for in other species. Even though such data are necessarily incomplete, because the same syntenic region will not have been assembled in each genome, this approach allowed us to polarise the evolutionary gains and losses of homeobox loci (Table 2, Figure 15). For example, if a locus is present in human but not mouse, and it is then found at the syntenic location in the dog genome, this allows us to deduce that the locus has been lost from the mouse genome; it is not a new gene arising somewhere on the evolutionary lineage leading to human. This logic is possible because the phylogenetic relationships between major mammalian orders are well established, based on extensive molecular data [29-31]. In the example given, because human is more closely related to mouse (in the Supraprimate clade, also called Euarchontoglires) than it is to dog (in Laurasiatheria), the gene must have existed in the common ancestor of Supraprimates and Laurasiatheria, which also includes the ancestry of the mouse lineage.

Considering gains of genes, there are parallels between the mouse and human evolutionary histories. The gene *LEUTX *seems to be a gain on the evolutionary lineage leading to human, and thus far we have not detected it outside primates. Since it contains a homeobox sequence, it must have originated from another homeobox gene by sequence divergence, probably preceded by gene duplication. The source gene is not known, although the location of *LEUTX *in chromosome band 19q13 suggests that *LEUTX *may have originated by tandem duplication from the *CRX *gene in the Otx gene family [3], or from the mammalian *TPRX1 *gene which itself may have originated from CRX. Interestingly, the gene gains in the rodent lineage map to the equivalent region of the mouse genome, although not in precisely the same syntenic location as *LEUTX*. Mouse *Crxos1 *is the immediate neighbour of the mouse *Crx *gene, while the Obox array, Gm5585 and Gm7235 lie on the other side of *Crx*. Two possible scenarios could account for this pattern. In one model, the Tprx loci (which are neighbours of *Crx *and existed in ancestral placental mammals) underwent extensive sequence divergence and then duplication in rodent evolution, generating the *Crxos1*, Obox genes, Gm5585 and Gm7235, while the same gene (or *Crx*) duplicated to give *Leutx *in primate evolution. Alternatively, the *Tprx1 *gene was lost by deletion in rodents, and the *Crxos1*, Obox, Gm5585 and Gm7235 genes arose directly by duplication and divergence of *Crx*. It is not yet possible to distinguish between these scenarios, although both highlight genomic plasticity of this region in both rodent and primate evolution.

Perhaps the most striking finding from our study relates to gene loss. By applying a phylogenomic approach, we found that the rodent lineage has experienced much more homeobox gene loss than has the primate lineage, in the same period of evolutionary time. The human genome has lost just a single homeobox gene, *Msx3*, whereas the mouse genome has secondarily lost *Ventx, Argfx, Dprx, Shox, Rax2, LOC647589, Tprx1 *and *Nanognb*. In seven of these cases the loss is by deletion of the gene or genomic region; the *Tprx1 *gene, discussed above, may be loss by deletion or simply by excessive divergence. It is not clear why gene loss should be so much more prevalent in one mammalian lineage than in another. There are, however, parallels elsewhere in the animal kingdom. A comparison of homeobox gene diversity in each of the three chordate subphyla - vertebrates, cephalochordates and tunicates - revealed dramatically different patterns of gene loss in each lineage. Starting from just over one hundred homeobox genes inferred to have been present in the common ancestor of all chordates, the tunicates (or at least those examined thus far) have lost 28 genes, vertebrates lost 9 genes (and duplicated others), while cephalochordates, represented by amphioxus *Branchiostoma floridae*, lost none [32].

## Conclusions

Resolving the complete patterns of gene gain and gene loss across the animal kingdom is an important goal for comparative genomics, and is relevant to any attempt to relate genome evolution to phenotypic evolution. In this research, we have started this line of enquiry for the mammalian homeobox genes, and uncovered an unexpected difference in the extent of gene loss between two evolutionary lineages.

Using comparative genomics, we find that there has been much more homeobox gene loss in the rodent evolutionary lineage than in the primate evolutionary lineage, since the time of divergence from their common ancestor. While the human lineage has lost only the *Msx3 *gene, mice have lost *Ventx, Argfx, Dprx, Shox, Rax2, LOC647589, Tprx1 *and *Nanognb*. This analysis provides insight into the patterns of homeobox gene evolution in the mammals, and is a step towards relating genomic evolution to phenotypic evolution.

As more mammalian genomes are sequenced to high coverage, assembled and annotated, it is hoped that further such studies will uncover the patterns and processes underlining genome evolution in this important and diverse taxon.

## Methods

Genome sequence data were downloaded from the NCBI FTP server[33], including *Homo sapiens *Build 37.2 (GRCh37.p2), *Mus musculus *Build 37.1 (C57BL/6J), *Pan troglodytes *Build 2.1 (Pan_troglodytes-2.1), *Macaca mulatta *Build 1.2 (Mmul_051212), *Rattus norvegicus *Build 4.2 (RGSC_v3.4), *Canis lupus familiaris *Build 2.1 (Dog2.0), *Bos Taurus *Build 5.2 (Btau_4.2), *Equus caballus *Build 2.1 (EquCab2), *Sus scrofa *Build 2.1 (Sscrofa9.2), *Gallus gallus *Build 2.1 (Gallus_gallus-2.1) and *Xenopus (Silurana) tropicalis *Build 1.1 (v4.2) (Table 2). These genomic sequences were first translated into peptides in all six possible reading frames using the 'transeq' tool in the EMBOSS suite [34]. These translated sequences constituted the target databases for homeodomain searches.

Searching for homeodomains employed the tools 'hmmbuild' and 'hmmsearch' in the HMMER3 package http://hmmer.org/[35] as follows. First, homeodomain sequences were retrieved from HomeoDBv1.2, using the 'download' tool, for the following species: human, amphioxus, beetle, fruitfly and honeybee [28]. These comprise comprehensive, manually-curated, datasets for each species. Second, ClustalW [36] was used to align homeodomain sequences, and this alignment converted manually into 'STOCKHOLM 1.0' format, as described in the 'User's Guide' for HMMER3. Third, hmmbuild was used to construct a profile hidden Markov model (profile HMM); and fourth, domain scanning used hmmsearch with the profile HMM and the translated genome sequences as inputs. The predicted homeodomain sequences from each species were collected manually from the HMMER3 search results files, and individually verified using BLAST toolkit [37] and CD-Search [38] before a final list of homeodomains was compiled. In cases of extreme sequence divergence, Phyre [39] was used to evaluate potential secondary structure. Gene annotation information from NCBI was then used to locate each predicted homeodomain in the genome sequence.

## Authors' contributions

YFZ carried out the genome analyses and contributed to interpretation of the results. PWHH conceived the study and contributed to interpretation of results. Both authors wrote and approved the manuscript.

## Supplementary Material

Additional file 1 **All human homeobox genes and pseudogenes**: classification, chromosomal location, homeodomain sequence, database identification numbers and synonyms. Click here for file

Additional file 2 **Comparison of human and mouse homeobox gene repertoires classified by gene family**. Click here for file

Additional file 3 **All mouse homeobox genes and pseudogenes**: classification, chromosomal location, homeodomain sequence, database identification numbers and synonyms. Click here for file

Additional file 4 **Unannotated mouse homeobox loci**: chromosomal location, accession number, homeodomain sequence and similarity. Click here for file

Additional file 5 ***LEUTX *orthologues in human, chimpanzee and macaque**. (A) Alignment of homeodomains showing high sequence conservation. (B) Syntenic chromosomal regions around *LEUTX *genes. Click here for file

Additional file 6 **Refined structure of *TPRX1 *gene at human chromosome 19q13.33**. (A) The *TPRX1 *locus is incorrectly annotated in the NCBI and EBI assemblies with a truncation at the 5' end. This predicts an incomplete homeodomain, even though the entire homeobox region is present in the genome sequence. (B) Revised gene model for *TPRX1 *based on cDNA data in GenBank, accessions AK097640, BC137501, BC144673, BC141863, DQ340180. Additional 5' exons are present, predicting a complete homeodomain sequence. Click here for file
