# Correction: The dynamics of vertebrate homeobox gene evolution: gain and loss of genes in mouse and human lineages

**DOI:** 10.1186/1471-2148-11-204

**Published:** 2011-07-13

**Authors:** Ying-fu Zhong, Peter WH Holland

**Affiliations:** 1 Department of Zoology, University of Oxford, South Parks Road, Oxford, OX1 3PS, UK

## Correction

After publication of this work [1], we found evidence for a gene (or two tandem genes) orthologous to *Leutx *in a genome assembly for the African elephant produced by the Broad Institute and accessible on the UCSC Genome Browser, assembly version Broad/loxAfr3 [2]. Elephant *Leutx *homeodomain sequences are highly divergent from that of human *LEUTX *(48% identity), but regions of identity outside the homeodomain plus genomic synteny (Figure 1) confirm orthology. We also found divergent putative *Leutx *orthologues in some other mammals including dog, cat, horse, pig and cow but, as reported in our original publication, not in rodents or outside the placental mammals. These findings imply that *Leutx *did not originate in primate evolution as we originally suggested [1], but arose early in the radiation of placental mammals and was secondarily lost from rodents. This finding strengthens our original conclusion that rodents have experienced far more homeobox gene loss than have primates. *Leutx *is added to *Ventx, Argfx, Dprx, Shox, Rax2, LOC647589, Tprx1 *and *Nanognb *on the list of homeobox genes lost in the evolutionary lineage leading to rodents (Figure 2).

**Figure 1:**
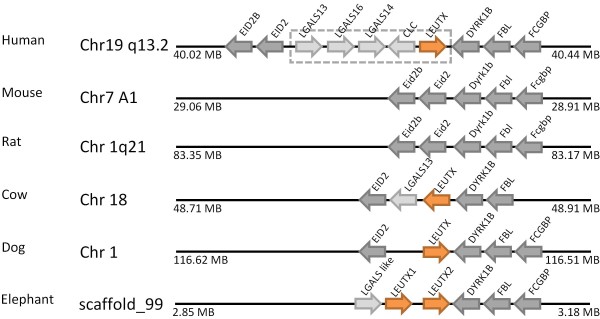
**The *LEUTX *homeobox gene (orange arrow) plus four unusual non-homeobox genes (light grey arrows) form a linked set of genes in the human genome (dashed box)**. Divergent putative *Leutx *genes are located in syntenic regions of cow, dog and elephant (orange arrows), but absent at the equivalent location in mouse and rat. Non-homeobox genes used as indicators of chromosomal synteny are shown as grey arrows.

**Figure 2:**
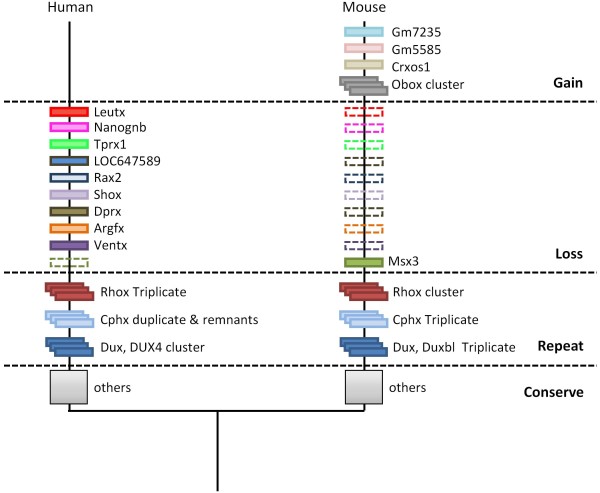
**A summary of homeobox gene dynamics in the mouse and human evolutionary lineages**. The majority of homeobox genes are conserved between mouse and human lineages (grey squares), although some have undergone duplication to different extents (cascaded boxes). Humans have lost the *Msx3 *gene; mice have lost *Leutx, Nanognb, Tprx1, LOC647589, Rax2, Shox, Dprx, Argfx *and *Ventx *(dashed boxes). Three new homeobox loci (*Gm7235, Gm5585 *and *Crxos1*) and one new cluster (Obox) arose in the rodent lineage.
