# Supplementary material for: The dynamics of vertebrate homeobox gene evolution: gain and loss of genes in mouse and human lineages
Source: BMC Evol Biol. 2011 Jun 16;11:169. doi: 10.1186/1471-2148-11-169 (PMC3141429; doi:10.1186/1471-2148-11-169)
Supplement: Additional file 5 — LEUTX orthologues in human, chimpanzee and macaque. (A) Alignment of homeodomains showing high sequence conservation. (B) Syntenic chromosomal regions around LEUTX genes. [file 1471-2148-11-169-S5.PPT]

## Slide 1
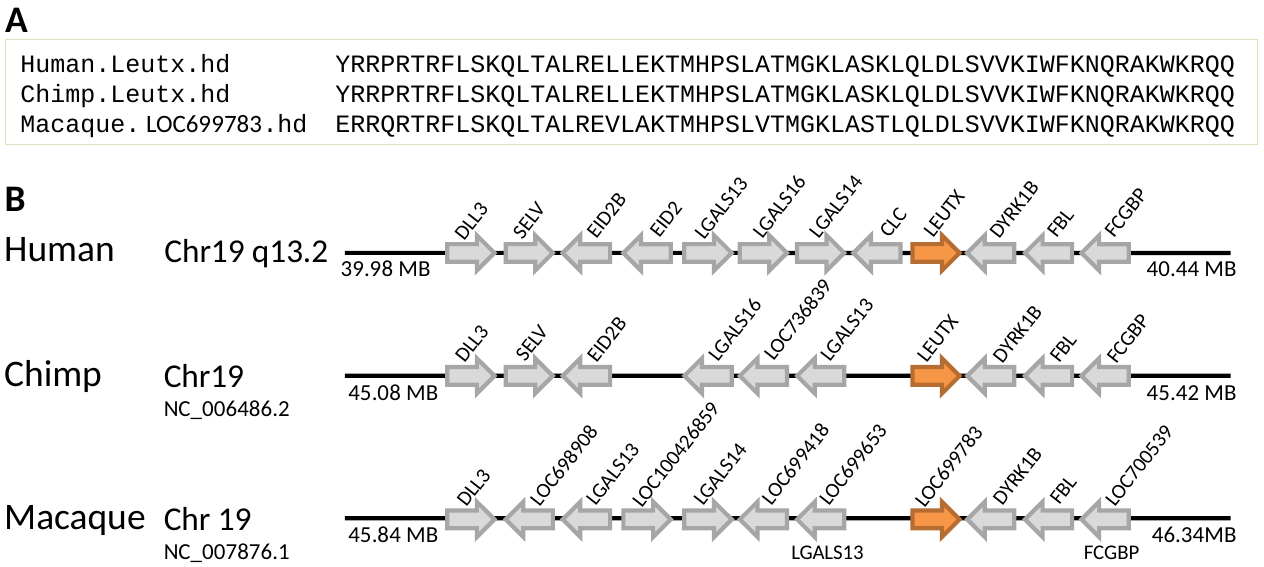

A
Human.Leutx.hd	 YRRPRTRFLSKQLTALRELLEKTMHPSLATMGKLASKLQLDLSVVKIWFKNQRAKWKRQQ
Chimp.Leutx.hd	 YRRPRTRFLSKQLTALRELLEKTMHPSLATMGKLASKLQLDLSVVKIWFKNQRAKWKRQQ
Macaque. LOC699783.hd	 ERRQRTRFLSKQLTALREVLAKTMHPSLVTMGKLASTLQLDLSVVKIWFKNQRAKWKRQQ
B
LGALS16
LGALS14
LGALS13
DYRK1B
FCGBP
LEUTX
EID2B
EID2
DLL3
SELV
CLC
FBL
Human
Chr19 q13.2
39.98 MB
40.44 MB
LOC736839
LGALS13
LGALS16
DYRK1B
LEUTX
FCGBP
EID2B
DLL3
SELV
FBL
Chimp
Chr19
NC_006486.2
45.08 MB
45.42 MB
LOC100426859
LOC699418
LOC699653
LOC698908
LOC700539
LOC699783
LGALS13
LGALS14
DYRK1B
DLL3
FBL
Macaque
Chr 19
NC_007876.1
45.84 MB
46.34MB
LGALS13
FCGBP
