# Supplementary material for: The dynamics of vertebrate homeobox gene evolution: gain and loss of genes in mouse and human lineages
Source: BMC Evol Biol. 2011 Jun 16;11:169. doi: 10.1186/1471-2148-11-169 (PMC3141429; doi:10.1186/1471-2148-11-169)
Supplement: Additional file 6 — Refined structure of TPRX1 gene at human chromosome 19q13.33. (A) The TPRX1 locus is incorrectly annotated in the NCBI and EBI assemblies with a truncation at the 5' end. This predicts an incomplete homeodomain, even though the entire homeobox region is present in the genome sequence. (B) Revised gene model for TPRX1 based on cDNA data in GenBank, accessions AK097640, BC137501, BC144673, BC141863, DQ340180. Additional 5' exons are present, predicting a complete homeodomain sequence. [file 1471-2148-11-169-S6.PPT]

## Slide 1
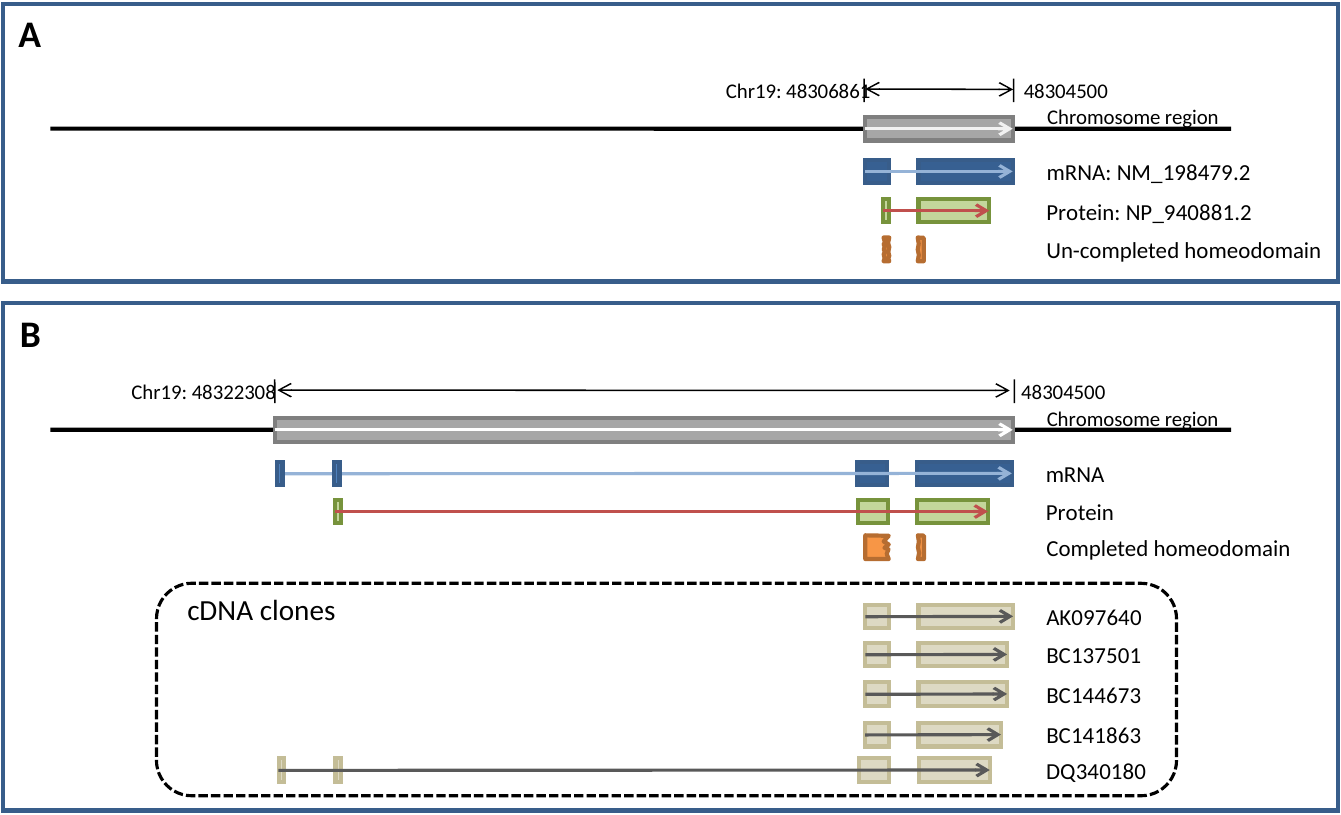

A
Chr19: 48306861
48304500
Chromosome region
mRNA: NM_198479.2
Protein: NP_940881.2
Un-completed homeodomain
B
Chr19: 48322308
48304500
Chromosome region
mRNA
Protein
Completed homeodomain
cDNA clones
AK097640
BC137501
BC144673
BC141863
DQ340180
